# Assessing the Land Reclamation Suitability of Beam Fabrication and Storage Yard in Railway Construction: An AHP-MEA Method

**DOI:** 10.3390/ijerph20053805

**Published:** 2023-02-21

**Authors:** Baoquan Cheng, Jianchang Li, Jingfang Tao, Jianling Huang, Huihua Chen

**Affiliations:** 1 School of Civil Engineering, Central South University, Changsha 410083, China; 2 Department of Architecture and Civil Engineering, City University of Hong Kong, Hong Kong 999077, China; 3 Imperial College Business School, Imperial College London, London SW7 2AZ, UK

**Keywords:** land reclamation suitability, beam fabrication and storage yard, sustainable railway, analytic hierarchy process, matter-element analysis

## Abstract

Railway construction contributes to socio-economic development but causes the occupation and destruction of land resources. How to effectively restore the temporary land and achieve efficient and rational reuse therefore becomes particularly important. The beam fabrication and storage yard (BFSY), as a large temporary facility during railway construction, occupies a large area of land. However, BFSYs damage the land in the way of pressing and may harden the ground to a high degree due to the use of high-density pile foundations, adversely affecting the soil properties. Therefore, this research aims to develop a model for evaluating the land reclamation suitability (LRS) of BFSY. The LRS evaluation indicator system of BFSY was firstly constructed based on the literature review and expert interviews. Then, an indicator-based model for assessing the LRS of BFSY was developed by integrating the analytic hierarchy process (AHP) model and the matter-element analysis (MEA) model. A case project in China was chosen to demonstrate and validate the developed model, and results show that the proposed model can rationally evaluate the LRS of BFSY in railway construction. The findings of this research enrich the knowledge system of sustainable railway construction and guide construction managers to conduct practical suitability assessments of land reclamation.

## 1. Introduction

According to the World Bank, as of 2016, the total length of global railways reached 1.05 million km worldwide, providing high-quality transportation services for passengers and goods. Railways perform an essential role in transport due to their high carrying capacity [1]. The rapid expansion of railways significantly contributes to economic growth, social development, and cultural communications [2,3]. In addition, improvements to the railway infrastructure in terms of accessibility and coverage are regarded as an essential component of a sustainable transport system [4]. This is because railways are considered an environmentally friendly way to efficiently transport millions of passengers and tons of goods across countries and continents while consuming less energy and cutting carbon emissions [5]. However, the sustainability of railway transportation often comes under question because of its environmental impact during infrastructure construction [6]. Developing railway transportation requires constructing a large amount of infrastructure, which occupies land, consumes energy and materials, emits carbon emissions, generates solid wastes, and causes many other environmental problems [7,8]. Thus, when discussing the sustainability of railway transportation, environmental impacts during infrastructure construction cannot be ignored.

Railway construction occupies and damages a huge number of land resources, of which 58.4% are caused by various temporary infrastructures such as waste disposal areas, pioneer roads, beam fabrication and storage yards (BFSYs), etc. [9,10]. Among these temporary infrastructures, BFSYs cover a larger area (usually about ten ${\mathrm{hm}}^{2}$). BFSYs damage the land in the way of pressing. In addition, during BFSY construction, high-density pile foundations are usually adopted, and the ground hardening degree is high. This results in the loss of the original topsoil, adversely affecting the soil properties and even damaging the surrounding roads and water systems. Conducting land reclamation of BFSYs appropriately is, therefore, necessary to reduce environmental impacts and improve the sustainability of railway construction.

The land suitability assessment is a foundation for land reclamation, which has drawn the attention of global scholars. For example, Amirshenava and Osanloo [11] established a general semi-quantitative method based on the 2D assessment matrix to evaluate the suitability of mine reclamation. Wang et al. [12] adopted an ecosystem service evaluation approach to provide a systematic framework for mine reclamation assessment. Yu et al. [13] used the fuzzy comprehensive evaluation model to assess land reclamation and constructed a comprehensive evaluation model to estimate the overall benefits of land reclamation. These studies provide diverse insights into assessing land reclamation suitability (LRS). However, previous studies usually investigated the LRS of mines, transformer substations, and other lands [11,14]. Research focusing on the LRS of BFSY is still unavailable. On the other hand, land reclamation of BFSY during railway construction is not mature. Due to limitations of cost, schedule, technology, and other factors, problems, such as soil quality decline and land degradation, often emerge after reclamation. Research on the LRS of BFSY is therefore urgently needed.

To address the research gap, this study aims to construct an indicator-based model for systematically and thoroughly evaluating the LRS of BFSY in railway construction by integrating the analytic hierarchy process (AHP) model and the matter-element analysis (MEA) model. The AHP model is used to determine the weight of each selected indicator, and the MEA model is used to assess the LRS of BFSY. Krmac and Djordjević used the AHP method to evaluate the impacts of train control information systems and key performance themes for sustainable railways [15]. However, this research has one limitation since the evaluation was conducted through the qualitative judgements. Bao and Qiu constructed a vulnerability evaluation system for the Sichuan-Tibet Railway and determined the weights of relevant assessment indicators through MEA [16]. However, integrating AHP and MEA can obtain more efficient evaluation results. For instance, Wang et al. proposed a demonstration model for selecting the most appropriate geophysical methods based on AHP and MEA [17]. In their research, the integrated model can reduce the impact of subjective factors to a certain extent, contributing to the practicality and cost effectiveness of geophysical surveys. Therefore, this study integrated AHP and MEA to evaluate the LRS of BFSY in railway construction.

A case project in China is adopted to validate the established model. The proposed approach can provide guidance to builders, governments, and engineers to clarify the direction of land reclamation utilization and provide them with valuable policy recommendations and technical standards in the actual implementation process, thus reducing the negative impact on the environment during railway construction.

The rest of this paper is organized as follows. Section 2 develops an AHP-MEA model for evaluating the LRS of BFSY. Section 3 demonstrates and validates the constructed model through a case study in China. Section 4 discusses the essential factors and implementation measures of the case project. Eventually, the conclusions are drawn in Section 5, and the limitations of the model are discussed in this section.

## 2. Methodology

**Figure 1:**
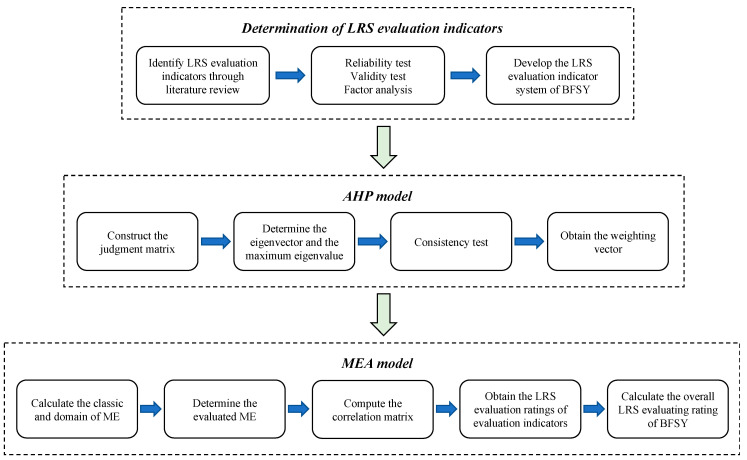
Methodology framework for evaluating the LRS of BFSY in railway construction.

According to the previous discussion, Figure 1 illustrates the framework of the indicator-based evaluation method for the LRS of BFSY in railway construction based on AHP-MEA. Firstly, the LRS evaluation indicator system of BFSY is constructed based on previous studies and screening of evaluation indicators through expert interviews. Secondly, the AHP model is applied to determine the weight of each LRS evaluation indicator. Then, the LRS evaluation rating of each indicator and the overall LRS rating of BFSY can be calculated by the MEA model. The detailed implementation process of this method is described in the following subsections.

### 2.1. Identification of LRS Evaluation Indicators

A comprehensive and reasonable evaluation indicator system is the foundation for accurately assessing the LRS of BFSY in railway construction. This research mainly collects and identifies LRS evaluation indicators from five dimensions through previous studies, which are listed in Table 1. When conducting land reclamation, attention should be paid to the carrying capacity of the resources and environment, and the natural factors of the area where the project is located should be fully considered to promote the improvement of the regional ecological environment. The ease of reclamation also deserves to be considered since extremely unreasonable inputs should not be generated for reclamation. The ease of reclamation should be evaluated to achieve a balance of reclamation inputs, direction, effectiveness, and difficulty. In addition, the location factors should be considered comprehensively to prevent inefficient land use issues caused by poor decision-making. Indeed, the public may be greatly concerned about location factors [10]. For example, if the site is located in an area prone to geological hazards, then reclamation will be considerably more difficult. Besides, in order to better utilize the social benefits of land reclamation, its impacts on the residents around the project site should be fully accounted for. Furthermore, the economic factor is essential as the cost and economic benefits should be considered to achieve the economic sustainability of land reclamation. Therefore, the five main dimensions for evaluating the LRS of BFSY are natural factors, ease of reclamation, location factors, social factors, and economic factors.

Furthermore, to ensure the rationality of the evaluation indicators, the initially selected evaluation indicators were reviewed through questionnaire surveys to establish the final LRS evaluation indicator system of BFSY. The five-level Likert scale was used to determine the importance of each evaluation indicator. The questionnaire was distributed to 30 experts in railway engineering (including 19 senior engineers from designing institutes, construction companies, and railway companies and 11 professors from universities), and each respondent was asked to rate the importance of each LRS evaluation indicator. These experts aged 40–54 years all have at least seven years of work experience in land reclamation projects, which ensures that surveyed experts have a deep understanding of evaluating the LRS of BFSYs. The data quality of the questionnaire directly affects the analysis and screening of indicators. Therefore, the reliability and validity of the questionnaire should be tested before the factor analysis. Firstly, in order to ensure the stability and consistency of the indicator scales in the questionnaire, Cronbach’s alpha was applied to test the reliability of the questionnaire. For Cronbach’s alpha, a value of 0.6 or less indicates poor reliability, a value between 0.6 and 0.7 indicates acceptable reliability, a value between 0.7 and 0.8 indicates great reliability, and a value greater than 0.8 indicates high reliability. In this study, the reliability test was conducted by SPSS 28 software (IBM, New York, NY, USA) in terms of both overall and five evaluation dimensions, and the results are shown in Table 2. It can be found that the overall and five evaluation dimensions have Cronbach’s alpha values greater than 0.7, which means that they have high reliability and can be used for further analysis.

After the reliability test, the validity test was conducted to determine whether the questionnaire data were suitable for factor analysis. This research applied the Kaiser–Meyer–Olkin (KMO) test and Bartlett’s sphericity test to make a judgment. In general, the closer the KMO value is to 1, the higher the correlation between the scores of the indicators and the more suitable the data is for factor analysis. When the KMO value is less than 0.5, factor analysis is not suitable. Table 3 illustrates the results of the validity test. The KMO value is 0.779, and the *p*-value of Bartlett’s sphericity test is 0.000, suggesting that the questionnaire data are suitable for factor analysis.

In order to determine the appropriate number of common factors to be extracted, it is necessary to conduct an analysis using the variance explanation ratio table. Factors whose eigenvalues are greater than one were generally selected as common factors based on the explanatory rates of the original variables. In this study, the 26 LRS evaluation indicators in the questionnaire were analyzed. The results indicate that the eigenvalues of the first 5 components are greater than 1, and the cumulative variance contribution rate of the first 5 components is 73.213%, which can reflect most of the information of the 26 indicators. Therefore, it can be judged that 5 principal components can be extracted from the 26 LRS evaluation indicators. Then, the questionnaire data were processed using factor analysis in order to categorize the 26 LRS evaluation indicators. The indicators with loadings greater than 0.5 within the same common factor were grouped together. According to the rotated component matrix (see Table 4), the ground levelness and geological hazard susceptibility fail to reach 0.5, which do not meet the convergent validity criteria and are excluded. In addition, the effective soil layer thickness and organic matter content should have belonged to component 3, but the calculation results show that they belong to component 1, so they are also excluded. Moreover, the pile foundation density should belong to component 5, and should not be classified as belonging to component 2 as shown in the software calculation results, so it is excluded.

**Figure 2:**
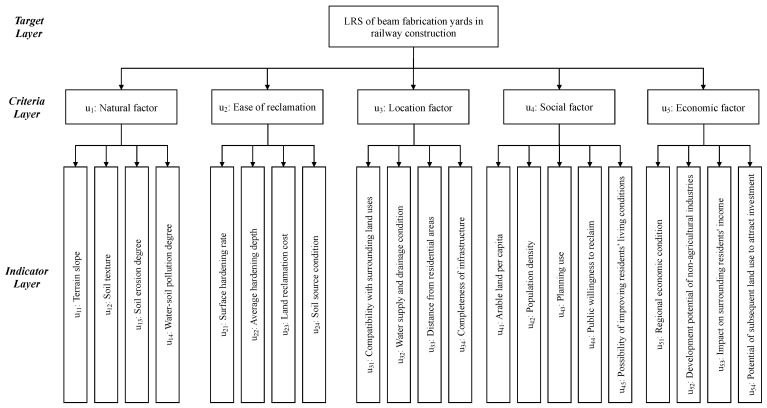
LRS evaluation indicator system of BFSY in railway construction.

Therefore, the final LRS evaluation indicator system of BFSY in railway construction is illustrated in Figure 2. This system consists of a target layer, a criteria layer, and an indicator layer. Among them, the target layer is the LRS of BFSY in railway construction. The criteria layer includes five criteria: natural factor, ease of reclamation, location factor, social factor, and economic factor. The indicator layer is composed of 21 LRS evaluation indicators.

### 2.2. AHP-Based Weight Calculation

The AHP model is a multilevel weighting decision approach proposed by Saaty [52]. It is useful to make decisions consisting of several interrelated and sometimes conflicting criteria, and to formulate priorities among decision criteria under decision objectives [53]. For example, Sang et al. integrated the AHP method, Delphi method, and GIS to assess the value of railway heritage landscape along the railway [54]. This research aims to evaluate the LRS of BFSY in railway construction, which can be implemented by the constructed LRS evaluation indicator system in Section 2.1. Thus, the AHP model can be applied to the determination of the weights of each LRS evaluation indicator and criterion. The main advantage of AHP is that it can help to check and reduce the inconsistency of expert judgment [55]. Therefore, the AHP model has been introduced by many scholars when assessing the suitability of land reclamation [56,57,58].

The first step in applying the AHP model is to construct the judgment matrix. As expressed in Equation (1), the judgment matrix Q for a set of evaluation indicators consists of the average comparative importance of each two evaluation indicators $q_{ij}$ to the criteria layer.

(1) $$Q={\left[q_{ij}\right]}_{n\times n}=\left[\begin{array}{cccc} q_{11} & q_{12} & \ldots & q_{1n}\\ q_{21} & q_{22} & \ldots & q_{2n}\\ \vdots & \vdots & \ddots & \vdots \\ q_{n1} & q_{n2} & \ldots & q_{nn} \end{array}\right]$$

where $q_{ij}$ is the average importance of the *i*-th evaluation indicator to the criteria layer compared to the *j*-th evaluation indicator, and it is the mean of $q_{ij}^{k}$ (Equation (2)) that is assessed by experts (Table 5).

(2) $$q_{ij}=\frac{1}{k}\overset{m}{\underset{k}{\sum }}q_{ij}^{k}$$

where n represents the number of evaluation indicators and m is the number of experts.

Then, the eigenvector $w_{i}$ and the maximum eigenvalue $\lambda_{max}$ can be determined through Equations (3) and (4), respectively:

(3) $$w_{i}=\frac{\sqrt[{n}]{\prod_{j=1}^{n}q_{ij}}}{\sum_{i=1}^{n}\sqrt[{n}]{\prod_{j=1}^{n}q_{ij}}}$$

(4) $$\lambda_{max}=\frac{1}{n}\overset{n}{\underset{i=1}{\sum }}\frac{{\left(QW\right)}_{i}}{w_{i}}$$

where i is the number of rank of Q and j is the number of column of Q.

Next, the consistency of the judgment matrix should be examined by the consistency ratio [20] to diminish the errors caused by the complexity of the system and experts’ biased preferences. The *CR* can be calculated through Equations (5) and (6):

(5) $$CI=\frac{\lambda_{max}-n}{n-1}$$

(6) $$CR=\frac{CI}{RI}$$

where CI represents the consistency index of the judgment matrix and RI denotes the average random consistency index of the judgment matrix.

The RI values of the judgment matrix with low dimension are illustrated in Table 6 [59].

If the RI value is within the limit of the consistent region of 0.1, the eigenvectors of the matrix can be considered as weights.

### 2.3. MEA-Based LRS Evaluation

To establish the LRS evaluation model for BFSY in railway construction, the MEA model is adopted to identify the correlation between different LRS evaluation indicators and to determine the LRS ratings. The MEA model is an evaluation method that effectively combines qualitative and quantitative analysis, which can fully reflect the overall level of the evaluated object. MEA was originally developed on the basis of extended sets to solve the incompatibility issues [60]. MEA applies mathematical methods such as topological sets and correlation functions to better address incompatible problems. By determining the matter-element (ME), classical domain, and joint domain, it is possible to calculate the correlation between each evaluation indicator. The higher the correlation, the more likely the indicator is to be at that level. Therefore, based on the AHP model, the MEA model can be used to develop the LRS evaluation model for BFSY in railway construction.

The ME is one of the essential constituent elements of topology, which is an ordered triplet unifying the interested matter, the attribute of the matter and the attribute value of the matter. Thus, the ME can be represented by Equation (7):

(7) R=(N, C, V)

where R denotes the ME; N refers to the interested matter; C is the attribute of N; and V represents the value of C.

In this study, the factor categories at the criteria layer are regarded as MEs, including the evaluation indicators under it at the indicator layer. The classic domain of the ME can be expressed in Equation (8):

(8) $$R_{c}^{n}(N^{n},C_{i}^{n},V_{ij}^{n})=\left[\begin{array}{ccccc} & L_{1} & L_{2} & \ldots & L_{j}\\ C_{1}^{n} & V_{11}^{n} & V_{12}^{n} & \ldots & V_{1j}^{n}\\ C_{2}^{n} & V_{21}^{n} & V_{22}^{n} & \ldots & V_{2j}^{n}\\ \vdots & \vdots & \vdots & \ldots & \vdots \\ C_{i}^{n} & V_{i1}^{n} & V_{i2}^{n} & \ldots & V_{ij}^{n} \end{array}\right]=\left[\begin{array}{ccccc} & L_{1} & L_{2} & \ldots & L_{j}\\ C_{1}^{n} & <a_{11}^{n},b_{11}^{n}> & <a_{12}^{n},b_{12}^{n}> & \ldots & <a_{1j}^{n},b_{1j}^{n}>\\ C_{2}^{n} & <a_{21}^{n},b_{21}^{n}> & <a_{22}^{n},b_{22}^{n}> & \ldots & <a_{2j}^{n},b_{2j}^{n}>\\ \vdots & \vdots & \vdots & \ldots & \vdots \\ C_{i}^{n} & <a_{i1}^{n},b_{i1}^{n}> & <a_{i2}^{n},b_{i2}^{n}> & \ldots & <a_{ij}^{n},b_{ij}^{n}> \end{array}\right]$$

where $R_{c}^{n}$ refers to the classic domain of the ME; $N^{n}$ is the *n*-th factor category; $C_{i}^{n}$ denotes the *i*-th evaluation indicator under $N^{n}$; $L_{j}$ represents the *j*-th rating criteria; and $\langle a_{ij}^{n},b_{ij}^{n}\rangle$ is the value interval of the rating criteria.

The total value domain of all ratings is the joint domain of the ME, which can be represented by Equations (9) and (10):

(9) $$R_{p}\left(N^{n},C_{i}^{n},V_{pi}^{n}\right)=\left[\begin{array}{ccc} N^{n} & C_{1}^{n} & V_{p1}^{n}\\ & C_{2}^{n} & V_{p2}^{n}\\ & \vdots & \vdots \\ & C_{i}^{n} & V_{pi}^{n} \end{array}\right]$$

(10) $$V_{pi}^{n}=\langle a_{pi}^{n},b_{pi}^{n}\rangle$$

where $R_{p}$ is the joint domain of the ME; $V_{pi}^{n}$ denotes the joint domain of $C_{i}^{n}$; and $V_{pi}^{n}=\langle a_{pi}^{n},b_{pi}^{n}\rangle$ refers to the value interval of joint domain.

Then, the evaluated ME can be represented by Equation (11):

(11) $$R_{0}\left(N_{0}^{n},C_{i}^{n},V_{0i}^{n}\right)=\left[\begin{array}{ccc} N^{n} & C_{1}^{n} & v_{01}^{n}\\ & C_{2}^{n} & v_{02}^{n}\\ & \vdots & \vdots \\ & C_{i}^{n} & v_{0i}^{n} \end{array}\right]\;$$

where $v_{0i}^{n}$ denotes the case value of $C_{i}^{n}$.

The correlation matrix measures the degree of correlation between an evaluation indicator and each evaluation level. Equations (12)–(15) show the process of correlation matrix calculation:

(12) $$K_{j}\left(v_{0i}^{n}\right)=\{\begin{array}{c} -\frac{\rho \left(v_{0i^{\prime }}^{n},V_{ij}^{n}\right)}{\left|V_{ij}^{n}\right|},\;\;\;\;\;\;\;\;\;\;\;\;\;\;\;\;v_{0i}^{n}\in V_{ij}^{n}\\ \frac{\rho \left(v_{0i^{\prime }}^{n}V_{ij}^{n}\right)}{\rho \left(v_{0i^{\prime }}^{n}V_{pi}^{n}\right)-\rho \left(v_{0i^{\prime }}^{n}V_{ij}^{n}\right)},\;\;\;v_{0i}^{n}\notin V_{ij}^{n} \end{array}$$

(13) $$\rho \left(v_{0i^{\prime }}^{n},V_{ij}^{n}\right)=\left|v_{0i}^{n}-\frac{a_{ij}^{n}+b_{ij}^{n}}{2}\right|-\frac{1}{2}\left(b_{ij}^{n}-a_{ij}^{n}\right)$$

(14) $$\rho \left(v_{0i^{\prime }}^{n},V_{pi}^{n}\right)=\left|v_{0i}^{n}-\frac{a_{pi}^{n}+b_{pi}^{n}}{2}\right|-\frac{1}{2}\left(b_{pi}^{n}-a_{pi}^{n}\right)$$

(15) $$\left|V_{ij}^{n}\left|=\right|a_{ij}^{n}-b_{ij}^{n}\right|$$

where $a_{ij}^{n}$ and $b_{ij}^{n}$ represent the minimum value and maximum value of the classical domain of *n*-th factor category, respectively; $a_{pi}^{n}$ and $b_{pi}^{n}$ represent the minimum value and maximum value of the joint domain of *n*-th factor category, respectively.

After that, the correlation matrix of each factor category $K_{j}\left(v_{0}^{n}\right)$ and the overall evaluation of LRS of BFSY in railway construction $K_{j}\left(v_{0}\right)$ can be computed through Equations (16) and (17), respectively:

(16) $$K_{j}\left(v_{0}^{n}\right)=\overset{m}{\underset{i=1}{\sum }}w_{i}^{n}\times K_{j}\left(v_{0i}^{n}\right)$$

(17) $$K_{j}\left(v_{0}\right)=\overset{n}{\underset{i=1}{\sum }}w^{n}\cdot K_{j}\left(v_{0}^{n}\right)$$

where $w_{i}^{n}$ is the weight of the *i*-th evaluation indicator under the *n*-th factor category; m refers to the number of the evaluation indicators under the *n*-th factor category; and $w^{n}$ denotes the weight of the *n*-th factor category.

After obtaining the correlation matrix, the rating of the evaluation indicators can be determined through Equations (18) and (19):

(18) $$\bar{K_{j}}\left(v_{0}\right)=\frac{K_{j}\left(v_{0}\right)-minK_{j}\left(v_{0}\right)}{\underset{j}{max}\left(v_{0}\right)-minK_{j}\left(v_{0}\right)}$$

(19) $$\bar{s}=\frac{\sum_{s=1}^{j}s\times \bar{K_{j}}\left(v_{0}\right)}{\sum_{s=1}^{j}\bar{K_{j}}\left(v_{0}\right)}$$

where $\bar{K_{j}}\left(v_{0}\right)$ is the normalized value of $K_{j}\left(v_{0}\right);$ and j refers to the number of rating criteria.

The LRS rating represents the degree of land reclamation suitability. The LRS with a lower rating indicates poor suitability for land reclamation. In this research, the LRS can be classified into four levels, and a detailed description of the levels is listed in Table 7.

## 3. Case Study

### 3.1. Background of Case Study

The LN high-speed railway is located in the south of Shandong Province, China, which is an essential east-west passenger corridor connecting the provincial capital cities with the regions along this line. The total length of this line is 138.284 km and there are 5 stations. The line is located in a hilly area with large undulations and complicated topographic conditions. The elevation of the line is 120–370 m, the relative height difference is 100–150 m, the terrain is undulating, and the topography is complex. Most of the land has a thin cover, with local cornerstones exposed and villages, farmland, and woodland developed. The soil along the project area is mainly brown loam, and the soil erodibility factor is relatively high. Thus, the soil erosion is prone to occur under the action of rainfall and wind. In the LN railway construction project, there are 6 BFSYs. Among them, the largest one is PY beam fabrication and storage yard (PYBFSY) with an area of 14.1086 ${\mathrm{hm}}^{2}$, which is selected as a case in this research. The soil along the PYBFSY is mainly brown loam with a surface layer thickness of about 20–30 cm. Moreover, the soil erodibility factor is high and soil erosion is likely to occur under rainfall and wind. In addition, the project area of the PYBFSY belongs to the Huaihe River basin, and the main rivers along the route are the Yi River, the Jun River, and the Altar River. The groundwater in the plain area is mainly distributed in the alluvial plain area of the Yi River and the Jun River and is mainly recharged by atmospheric precipitation and surface runoff. The groundwater is mainly pore water of the Fourth Series, and the major aquifer is the sand and gravel layer of the alluvial floodplain, which is rich in water.

### 3.2. Weight and Rating Criteria Determination

In this research, 30 experts mentioned in Section 2.1 were invited to assess the relative importance of each two evaluation indicators, and the judgment matrices was created based on the assessment results. After that, according to Equation (8), the weights of each evaluation indicator can be calculated. As shown in Table 8, Table 9, Table 10, Table 11, Table 12 and Table 13, judgment matrices were constructed for the evaluation indicators in the indicator layer and the factor categories in the criteria layer, and then their weights were calculated.

As shown in Table 8, Table 9, Table 10, Table 11 and Table 12, the *CR* of the weight judgment matrix of the indicator layer under each criterion is less than 0.1, which meets the requirement of the consistency test, and the results are acceptable. From Table 13, the *CR* of the weight judgment matrix of each criteria layer is also smaller than 0.1, indicating that the result is also acceptable.

Based on the descriptions of LRS ratings in Table 7 and experts’ recommendations, the rating criteria of the LRS evaluation indicators can be determined as illustrated in Table 14.

### 3.3. Classic Domain, Joint Domain and ME Determination

According to the rating criteria of evaluation indicators in Table 7, the classic domain $R_{i}\left(u_{i}\right)$ and the joint domain $R_{p}\left(u_{i}\right)$ of factor categories can be determined. The results of natural factor ($u_{1}$), ease of reclamation ($u_{2}$), location factor ($u_{3}$), social factor ($u_{4}$), and economic factor ($u_{5}$) are illustrated in Equations (20)–(24), respectively. As shown in Equation (25), the evaluated ME $R\left(u_{i}\right)$ can be calculated based on experts’ assessment.

(20) $$R_{1}\left(u_{1}\right)=\left[\begin{array}{ccc} N_{1} & u_{11} & \left[0,40\right)\\ & u_{12} & \left[0,25\right)\\ & u_{13} & \left[0,25\right)\\ & u_{14} & \left[0,25\right) \end{array}\right]\;\;\;\;\;\;\;R_{2}\left(u_{1}\right)=\left[\begin{array}{ccc} N_{2} & u_{11} & \left[40,65\right)\\ & u_{12} & \left[25,50\right)\\ & u_{13} & \left[25,50\right)\\ & u_{14} & \left[25,50\right) \end{array}\right]\;R_{3}\left(u_{1}\right)=\left[\begin{array}{ccc} N_{3} & u_{11} & \left[65,90\right)\\ & u_{12} & \left[50,75\right)\\ & u_{13} & \left[50,75\right)\\ & u_{14} & \left[50,75\right) \end{array}\right]\;\;\;\;\;\;\;R_{4}\left(u_{1}\right)=\left[\begin{array}{ccc} N_{4} & u_{11} & \left[90,100\right)\\ & u_{12} & \left[75,100\right)\\ & u_{13} & \left[75,100\right)\\ & u_{14} & \left[75,100\right) \end{array}\right]\;R_{p}\left(u_{1}\right)=\left[\begin{array}{ccc} N_{p} & u_{11} & \left[0,100\right)\\ & u_{12} & \left[0,100\right)\\ & u_{13} & \left[0,100\right)\\ & u_{14} & \left[0,100\right) \end{array}\right]$$

(21) $$R_{1}\left(u_{2}\right)=\left[\begin{array}{ccc} N_{1} & u_{21} & \left[0,40\right)\\ & u_{22} & \left[0,40\right)\\ & u_{23} & \left[0,25\right)\\ & u_{24} & \left[0,25\right) \end{array}\right]\;\;\;\;\;\;\;R_{2}\left(u_{2}\right)=\left[\begin{array}{ccc} N_{2} & u_{21} & \left[40,60\right)\\ & u_{22} & \left[40,60\right)\\ & u_{23} & \left[25,50\right)\\ & u_{24} & \left[25,50\right) \end{array}\right]\;R_{3}\left(u_{2}\right)=\left[\begin{array}{ccc} N_{3} & u_{21} & \left[60,90\right)\\ & u_{22} & \left[60,90\right)\\ & u_{23} & \left[50,75\right)\\ & u_{24} & \left[50,75\right) \end{array}\right]\;\;\;\;\;\;\;R_{4}\left(u_{2}\right)=\left[\begin{array}{ccc} N_{4} & u_{21} & \left[90,100\right)\\ & u_{22} & \left[90,100\right)\\ & u_{23} & \left[75,100\right)\\ & u_{24} & \left[75,100\right) \end{array}\right]\;R_{p}\left(u_{2}\right)=\left[\begin{array}{ccc} N_{p} & u_{21} & \left[0,100\right)\\ & u_{22} & \left[0,100\right)\\ & u_{23} & \left[0,100\right)\\ & u_{24} & \left[0,100\right) \end{array}\right]$$

(22) $$R_{1}\left(u_{3}\right)=\left[\begin{array}{ccc} N_{1} & u_{31} & \left[0,25\right)\\ & u_{32} & \left[0,25\right)\\ & u_{33} & \left[0,20\right)\\ & u_{34} & \left[0,25\right) \end{array}\right]\;\;\;\;\;\;\;R_{2}\left(u_{3}\right)=\left[\begin{array}{ccc} N_{2} & u_{31} & \left[25,50\right)\\ & u_{32} & \left[25,50\right)\\ & u_{33} & \left[20,60\right)\\ & u_{34} & \left[25,50\right) \end{array}\right]\;R_{3}\left(u_{3}\right)=\left[\begin{array}{ccc} N_{3} & u_{31} & \left[50,75\right)\\ & u_{32} & \left[50,75\right)\\ & u_{33} & \left[60,80\right)\\ & u_{34} & \left[50,75\right) \end{array}\right]\;\;\;\;\;\;\;R_{4}\left(u_{3}\right)=\left[\begin{array}{ccc} N_{4} & u_{31} & \left[75,100\right)\\ & u_{32} & \left[75,100\right)\\ & u_{33} & \left[80,100\right)\\ & u_{34} & \left[75,100\right) \end{array}\right]\;R_{p}\left(u_{3}\right)=\left[\begin{array}{ccc} N_{p} & u_{31} & \left[0,100\right)\\ & u_{32} & \left[0,100\right)\\ & u_{33} & \left[0,100\right)\\ & u_{34} & \left[0,100\right) \end{array}\right]$$

(23) $$R_{1}\left(u_{4}\right)=\left[\begin{array}{ccc} N_{1} & u_{41} & \left[0,25\right)\\ & u_{42} & \left[0,60\right)\\ & u_{43} & \left[0,25\right)\\ & u_{44} & \left[0,25\right)\\ & u_{45} & \left[0,25\right) \end{array}\right]\;\;\;\;\;\;R_{2}\left(u_{4}\right)=\left[\begin{array}{ccc} N_{2} & u_{41} & \left[25,50\right)\\ & u_{42} & \left[60,75\right)\\ & u_{43} & \left[25,50\right)\\ & u_{44} & \left[25,50\right)\\ & u_{45} & \left[25,50\right) \end{array}\right]\;R_{3}\left(u_{4}\right)=\left[\begin{array}{ccc} N_{3} & u_{41} & \left[50,75\right)\\ & u_{42} & \left[75,90\right)\\ & u_{43} & \left[50,75\right)\\ & u_{44} & \left[50,75\right)\\ & u_{45} & \left[50,75\right) \end{array}\right]\;\;\;\;\;\;R_{4}\left(u_{4}\right)=\left[\begin{array}{ccc} N_{4} & u_{41} & \left[75,100\right)\\ & u_{42} & \left[90,100\right)\\ & u_{43} & \left[75,100\right)\\ & u_{44} & \left[75,100\right)\\ & u_{45} & \left[75,100\right) \end{array}\right]\;R_{p}\left(u_{4}\right)=\left[\begin{array}{ccc} N_{p} & u_{41} & \left[0,100\right)\\ & u_{42} & \left[0,100\right)\\ & u_{43} & \left[0,100\right)\\ & u_{44} & \left[0,100\right)\\ & u_{45} & \left[0,100\right) \end{array}\right]$$

(24) $$R_{1}\left(u_{5}\right)=\left[\begin{array}{ccc} N_{1} & u_{51} & \left[0,10\right)\\ & u_{52} & \left[0,25\right)\\ & u_{53} & \left[0,25\right)\\ & u_{54} & \left[0,25\right) \end{array}\right]\;\;\;\;\;\;\;R_{2}\left(u_{5}\right)=\left[\begin{array}{ccc} N_{2} & u_{51} & \left[10,30\right)\\ & u_{52} & \left[25,50\right)\\ & u_{53} & \left[25,50\right)\\ & u_{54} & \left[25,50\right) \end{array}\right]\;R_{3}\left(u_{5}\right)=\left[\begin{array}{ccc} N_{3} & u_{51} & \left[30,50\right)\\ & u_{52} & \left[50,75\right)\\ & u_{53} & \left[50,75\right)\\ & u_{54} & \left[50,75\right) \end{array}\right]\;\;\;\;\;\;\;R_{4}\left(u_{5}\right)=\left[\begin{array}{ccc} N_{4} & u_{51} & \left[50,100\right)\\ & u_{52} & \left[75,100\right)\\ & u_{53} & \left[75,100\right)\\ & u_{54} & \left[75,100\right) \end{array}\right]\;R_{p}\left(u_{5}\right)=\left[\begin{array}{ccc} N_{p} & u_{51} & \left[0,100\right)\\ & u_{52} & \left[0,100\right)\\ & u_{53} & \left[0,100\right)\\ & u_{54} & \left[0,100\right) \end{array}\right]$$

(25) $$R\left(u_{1}\right)=\left[\begin{array}{ccc} N & u_{11} & 94\\ & u_{12} & 62\\ & u_{13} & 80\\ & u_{14} & 80 \end{array}\right]\;\;\;\;\;\;\;\;\;\;R\left(u_{2}\right)=\left[\begin{array}{ccc} N & u_{21} & 30\\ & u_{22} & 50\\ & u_{23} & 48\\ & u_{24} & 60 \end{array}\right]\;R\left(u_{3}\right)=\left[\begin{array}{ccc} N & u_{31} & 90\\ & u_{32} & 85\\ & u_{33} & 18\\ & u_{34} & 55 \end{array}\right]\;\;\;\;\;\;\;\;\;R\left(u_{4}\right)=\left[\begin{array}{ccc} N & u_{41} & 68\\ & u_{42} & 53\\ & u_{43} & 90\\ & u_{44} & 90\\ & u_{45} & 20 \end{array}\right]\;R\left(u_{5}\right)=\left[\begin{array}{ccc} N & u_{51} & 36\\ & u_{52} & 70\\ & u_{53} & 65\\ & u_{54} & 60 \end{array}\right]$$

### 3.4. Correlation Matrix Calculation and LRS Rating Determination

As listed in Table 15, the correlation matrix of each indicator at the indicator layer can be calculated according to Equations (12)–(17).

Furthermore, combining the weights of each evaluation indicator, the correlation matrix of each factor category at the criteria layer can be determined, as shown in Table 16. Moreover, the correlation matrix at the target layer can be calculated (Table 17).

Using Equations (18) and (19), the normalized LRS rating value of PYBFSY can be calculated: $\bar{s}=3.356$. Therefore, the LRS rating of PYBFSY is IV, suggesting that PYBFSY has the characteristics of high LRS. This means that the land reclamation potential of PYBFSY is high and corresponding measures can be taken.

## 4. Further Discussion

Among the factor categories at the criteria layer, the LRS ratings of the natural factor (u_1_), the location factor (u_3_), and the social factor (u_4_) are the highest, followed by the economic factor (u_5_) and the ease of reclamation (u_2_). The LRS rating of the ease of reclamation is II, which is barely suitable, indicating that this factor poses a significant barrier to land reclamation of PYBFSY. Indeed, the ease of reclamation fundamentally determines whether the land can be successfully reclaimed. For instance, if the ground is quite hard and has poor soil conditions, then this greatly increases the difficulty of reclamation, resulting in lower LRS [61]. Therefore, great attention should be focused on the ease of reclamation to promote the LRS rating of PYBFSY. In addition, although the LRS rating of the economic factor is better than that of the ease of reclamation, it is only moderately suitable at level III. In the reclamation of land, it is essential not to incur extremely unreasonable human, financial, and material costs to achieve the purpose of reclamation [62]. Instead, a balance should be achieved among reclamation investment, reclamation direction, reclamation effect, and reclamation difficulty. Thus, concern for the improvement of LRS by economic factors is also an important direction. All the LRS ratings of the natural factor, the location factor, and the social factor are IV, suggesting that they have high LRS in the PYBFSY.

In terms of the natural factor (u_1_), the LRS ratings of the terrain slope (u_11_), the soil erosion degree (u_13_), and the water-soil pollution degree (u_14_) are higher than that of the soil texture (u_12_). Thus, attention should be paid to the indicator of soil texture, and measures should be adopted to improve its LRS rating. Soil texture is a vital reflection of soil source conditions and plays an important role in promoting the effective realization of land reclamation effects [63]. Hence, it is possible to improve its LRS rating by reducing the damage to the soil texture during construction. For example, during the operation of PYBFSY, the combined effects of disturbances, such as the crushing of construction vehicles and the stockpiling of construction materials, can lead to compacted soils with poor permeability and aeration. Therefore, the land of PYBFSY should be tilled and loosened in time to enhance the level of drought and flood resistance so that the soil texture can be improved and the function of the land can be restored.

The ease of reclamation (u_2_) has the lowest LRS rating among the five factors at the criteria layer, and the LRS rating of the surface hardening rate (u_21_) is the worst among the four evaluation indicators related to the ease of reclamation. Accordingly, the focus should be placed on developing measures to promote the shift of its LRS rating to a higher direction. If the surface hardening is excessively deep, the soil structure will be severely damaged, thus aggravating the difficulty of reclamation [64]. Therefore, when demolishing the masonry in the PYBFSY, emphasis should be placed on removing the hardened floors. The BFSY consists of beam-making areas, beam storage areas, mixing areas, reinforcement sheds, and office and living areas. Among them, serious surface hardening exists in the beam-making area and the beam storage area, which can be broken and demolished using a crushing hammer combined with an excavator. Then, the excavator can be used to clear the surface gravel, remove the remaining garbage, stones, slag, etc., and fill the quarry pit in the surrounding villages. In addition, the LRS ratings of average hardening depth (u_22_) and land reclamation cost (u_23_) are not quite great. Therefore, when taking measures to reduce the hardening of the land, it is also important to control the costs involved so that they do not become excessive.

Regarding the location factor (u_3_), the LRS rating of the distance from residential areas (u_33_) is only I, which means that this indicator is a severe impediment to the LRS of PYBFSY. Therefore, the location of BFSY should be as far away from residential areas as possible. The reclamation area of BFSY is mainly located on both sides of the railroad line, so its reclamation should be coordinated with the construction and operation of the railroad [65]. While ensuring that the implementation of land reclamation measures does not interfere with the safety and normal operation of the railroad, it is necessary to create a channel with great visibility and environmental conditions to the extent possible. In addition, the location of BFSY should consider its effect on land use along the route, including adverse factors, such as noise, vibration, and land isolation along the route. As for the social factor (u_4_), both the LRS ratings of the population density (u_42_) and the possibility of improving residents’ living conditions (u_45_) are I. The BFSY in railway construction can have a large negative impact on the surrounding residents. Hence, when planning the BFSY construction, full consideration should be given to the local population density and the impact on the surrounding residents.

All four evaluation indicators under the economic factor (u_5_) are in the same LRS rating (III), which indicates that the economic factors have moderate LRS for PYBFSY. The expense of land reclamation is an essential component of the ease of reclamation, and it is also a key concern for the land reclamation of BFSY. In the implementation process, the deposit, management, use, and audit of funds should be standardized [66]. Firstly, for the deposit of land reclamation costs, the contractor should set up a special account for land reclamation costs and deposit all the land reclamation costs specified in the approved land reclamation plan into the account at one time. Secondly, land reclamation expense management should be implemented based on a joint management system developed by the contractor and the natural resources department with bank supervision. Therefore, the land reclamation expenses should be strictly in accordance with the principle of earmarking and not changing the use of the expenses at will. At the same time, the contractor should take the initiative to disclose the use of the land reclamation fee to the natural resources authority regularly. In addition, the project management system should be strictly implemented in the use of land reclamation expenses. Specifically, the system of in-service supervision and post-inspection should be implemented simultaneously. This will prevent the sloppy use of funds and their diversion to other uses. Eventually, regarding the audit of land reclamation expenses, the operation of land reclamation expenses should be regularly or irregularly inspected, and an accountability system should be implemented.

## 5. Conclusions

This research proposed an approach for assessing the LRS of BFSY in railway construction. An evaluation indicator system for LRS evaluation was developed on the basis of the previous literature. The influence factors of LRS can be classified into five categories: the natural factor, the ease of reclamation, the location factor, the social factor, and the economic factor. In addition, there are 21 LRS evaluation indicators under the five factor categories. A quantitative model for evaluating the LRS of BFSY in railway construction corresponding to the evaluation indicator system was also constructed by integrating the AHP and MEA, where AHP is used to determine the weight of each indicator and MEA to assess the LRS of BFSY.

To demonstrate and validate the proposed evaluation model, this research used PYBFSY in China for a case study. The results revealed that the LRS rating of PYBFSY is IV, indicating that PYBFSY has the characteristics of high LRS and corresponding measures can be taken based on the LRS rating. Among the factor categories at the criteria layer, the LRS ratings of the natural factor, the location factor, and the social factor were identified to be the highest, followed by the economic factor and the ease of reclamation. Furthermore, at the indicator layer, the surface hardening rate, the average hardening depth, the land reclamation cost, and the soil source condition were determined as key indicators to improve the LRS. Therefore, some land reclamation implementation measures were provided.

This study complements and enriches the knowledge body of sustainable railway construction. A quantitative evaluation model for the LRS of BFSY was developed for the first time for railway construction through constructing an evaluation indicator system and integrating AHP and MEA. The developed model can provide guidance to contractors, governments, site managers, and researchers in evaluating the LRS, helping them to identify key evaluation indicators and adopt effective land reclamation measures accordingly.

However, there are some limitations in this research: (1) Since this study only identifies evaluation indicators from previous literature, the selected evaluation indicators may be one-sided, so further exploration of the indicator system is needed. (2) This research only used one BFSY to validate the developed model. More empirical studies are needed to identify common factors in land reclamation of BFSY in railway construction. (3) Only Chinese cases were selected for this study, and in order for the model to be generalized to a wider context, cases from different countries should be selected for comparative analysis. Therefore, we recommend that future studies concentrate on the above-mentioned aspects.

## Figures and Tables

**Table 1:** Identification of LRS evaluation indicators through literature review.

Evaluation Dimension	Evaluation Indicator	Previous Studies
Natural factor	Terrain slope	[14,18]
	Effective soil layer thickness	[19]
	Soil texture	[20,21]
	Organic matter content	[22]
	Soil erosion degree	[23]
	Water-soil pollution degree	[24,25]
Ease of reclamation	Surface hardening rate	[26]
	Average hardening depth	[27]
	Pile foundation density	[28]
	Land reclamation cost	[29,30]
	Soil source condition	[31,32]
	Ground levelness	[33]
Location factor	Compatibility with surrounding land uses	[34]
	Water supply and drainage condition	[35,36]
	Distance from residential areas	[37]
	Completeness of infrastructure	[38]
	Geological hazard susceptibility	[39]
Social factor	Arable land per capita	[40]
	Population density	[41,42]
	Planning use	[43]
	Public willingness to reclaim	[44,45]
	Possibility of improving residents’ living conditions	[46]
Economic factor	Regional economic condition	[13]
	Development potential of non-agricultural industries	[11,47]
	Impact on surrounding residents’ income	[48,49]
	Potential of subsequent land use to attract investment	[50,51]

**Table 2:** Results of the reliability test.

Tested Variable	Number of Corresponding Indicator	Cronbach’s Alpha
Overall	26	0.944
Natural factor	6	0.751
Ease of reclamation	6	0.865
Location factor	5	0.807
Social factor	5	0.881
Economic factor	4	0.897

**Table 3:** Results of the validity test.

**KMO Value**		0.799
**Bartlett’s sphericity test**	Chi-squared value	2054.428
	Degree of freedom	325
	*p*-value	0.000

**Table 4:** Rotated component matrix.

Evaluation Indicator	Component 1	Component 2	Component 3	Component 4	Component 5
Terrain slope	0.264	0.231	0.745	0.235	−0.170
Effective soil layer thickness	0.587	0.463	0.284	0.047	0.191
Soil texture	0.101	0.218	0.726	−0.190	0.260
Organic matter content	0.896	−0.054	−0.101	−0.071	0.180
Soil erosion degree	0.017	0.039	0.819	0.220	−0.042
Water-soil pollution degree	−0.069	0.393	0.700	0.060	0.276
Surface hardening rate	0.433	0.294	−0.092	0.131	0.641
Average hardening depth	0.493	0.308	0.009	0.247	0.618
Pile foundation density	0.578	0.605	0.271	0.047	0.020
Land reclamation cost	0.272	0.043	0.542	0.210	0.550
Soil source condition	0.147	0.248	0.457	0.057	0.559
Ground levelness	0.470	0.297	0.430	0.210	0.476
Compatibility with surrounding land uses	0.159	0.140	0.383	0.685	−0.183
Water supply and drainage condition	0.265	0.173	−0.049	0.773	0.096
Distance from residential areas	0.071	0.186	0.293	0.631	0.410
Completeness of infrastructure	0.237	0.188	−0.070	0.711	0.489
Geological hazard susceptibility	−0.064	0.425	0.288	0.449	0.271
Arable land per capita	0.700	0.455	0.130	0.165	−0.021
Population density	0.727	0.223	0.026	0.234	0.266
Planning use	0.802	0.054	0.131	0.121	0.156
Public willingness to reclaim	0.708	−0.077	0.142	0.276	0.297
Possibility of improving residents’ living conditions	0.751	0.426	0.131	0.176	−0.070
Regional economic condition	0.471	0.678	0.085	0.220	0.130
Development potential of non-agricultural industries	0.029	0.739	0.249	0.086	0.383
Impact on surrounding residents’ income	0.108	0.842	0.216	0.274	0.216
Potential of subsequent land use to attract investment	0.400	0.624	0.271	0.301	0.077

**Table 5:** Scale of average importance.

Scale of Average Importance	Rating	Reciprocal
Equal importance	1	1
Weak or slight importance	2	1/2
Moderate importance	3	1/3
Moderate to strong importance	4	1/4
Strong importance	5	1/5
Strong to very strong importance	6	1/6
Very strong importance	7	1/7
Very strong to extreme importance	8	1/8
Extreme importance	9	1/9

**Table 6:** RI values of the judgment matrix with low dimension.

n	3	4	5	6	7	8	9
*RI*	0.58	0.90	1.12	1.24	1.32	1.41	1.45

**Table 7:** LRS ratings and their descriptions.

Rating	LRS Level	Description
I	Unsuitable	No compatibility with surrounding land use patterns.
II	Barely suitable	Large differences and poor compatibility with surrounding land use patterns.
III	Moderately suitable	Small differences and good compatibility with surrounding land use patterns.
IV	Highly suitable	Identical and compatible with surrounding land use patterns.

**Table 8:** The judgment matrix and weights of evaluation indicators under u_1_.

u_1_	u_11_	u_12_	u_13_	u_14_	Weight	Consistency Test
u_11_	1	2	3	3	0.4512	
u_12_	1/2	1	2	2	0.2609	$\lambda_{max}=4.0710$
u_13_	1/3	1/2	1	1/2	0.1190	CR=0.0266<0.1
u_14_	1/3	1/2	2	1	0.1689	

**Table 9:** The judgment matrix and weights of evaluation indicators under u_2_.

u_2_	u_21_	u_22_	u_23_	u_24_	Weight	Consistency Test
u_21_	1	1/2	3	2	0.2926	
u_22_	2	1	3	2	0.4155	$\lambda_{max}=4.0532$
u_23_	1/3	1/3	1	1/2	0.1070	CR=0.0172<0.1
u_24_	1/2	1/2	2	1	0.1849	

**Table 10:** The judgment matrix and weights of evaluation indicators under u_3_.

u_3_	u_31_	u_32_	u_33_	u_34_	Weight	Consistency Test
u_31_	1	2	4	5	0.5068	
u_32_	1/2	1	2	3	0.2641	$\lambda_{max}=4.0211$
u_33_	1/4	1/2	1	2	0.1428	CR=0.0079<0.1
u_34_	1/5	1/3	1/2	1	0.0863	

**Table 11:** The judgment matrix and weights of evaluation indicators under u_4_.

u_4_	u_41_	u_42_	u_43_	u_44_	u_45_	Weight	Consistency Test
u_41_	1	4	2	2	3	0.3703	
u_42_	1/4	1	1/2	1/3	2	0.1018	$\lambda_{max}=5.1388$
u_43_	1/2	2	1	1	4	0.2191	CR=0.0310<0.1
u_44_	1/2	3	1	1	4	0.2389	
u_45_	1/3	1/2	1/4	1/4	1	0.0699	

**Table 12:** The judgment matrix and weights of evaluation indicators under u_5_.

u_5_	u_51_	u_52_	u_53_	u_54_	Weight	Consistency Test
u_51_	1	2	3	4	0.4692	
u_52_	1/2	1	1/2	3	0.1942	$\lambda_{max}=4.1596$
u_53_	1/3	2	1	3	0.2524	CR=0.0598<0.1
u_54_	1/4	1/3	1/3	1	0.0842	

**Table 13:** The judgment matrix and weights of evaluation indicators under u.

u	u_1_	u_2_	u_3_	u_4_	u_5_	Weight	Consistency Test
u_1_	1	1	2	3	2	0.2977	
u_2_	1	1	1	3	2	0.2611	$\lambda_{max}=5.3239$
u_3_	1/2	1	1	1/2	2	0.1683	CR=0.0723<0.1
u_4_	1/3	1/3	2	1	1	0.1488	
u_5_	1/2	1/2	1/2	1	1	0.1191	

**Table 14:** Rating criteria of the LRS evaluation indicators.

Evaluation Indicator	I	II	III	IV
u_11_	[0, 40)	[40, 65)	[65, 90)	[90, 100)
u_12_	[0, 25)	[25, 50)	[50, 75)	[75, 100)
u_13_	[0, 25)	[25, 50)	[50, 75)	[75, 100)
u_14_	[0, 25)	[25, 50)	[50, 75)	[75, 100)
u_21_	[0, 40)	[40, 60)	[60, 90)	[90, 100)
u_22_	[0, 40)	[40, 60)	[60, 90)	[90, 100)
u_23_	[0, 25)	[25, 50)	[50, 75)	[75, 100)
u_24_	[0, 25)	[25, 50)	[50, 75)	[75, 100)
u_31_	[0, 25)	[25, 50)	[50, 75)	[75, 100)
u_32_	[0, 25)	[25, 50)	[50, 75)	[75, 100)
u_33_	[0, 20)	[20, 60)	[60, 80)	[80, 100)
u_34_	[0, 25)	[25, 50)	[50, 75)	[75, 100)
u_41_	[0, 25)	[25, 50)	[50, 75)	[75, 100)
u_42_	[0, 60)	[60, 75)	[75, 90)	[90, 100)
u_43_	[0, 25)	[25, 50)	[50, 75)	[75, 100)
u_44_	[0, 25)	[25, 50)	[50, 75)	[75, 100)
u_45_	[0, 25)	[25, 50)	[50, 75)	[75, 100)
u_51_	[0, 10)	[10, 30)	[30, 50)	[50, 100)
u_52_	[0, 25)	[25, 50)	[50, 75)	[75, 100)
u_53_	[0, 25)	[25, 50)	[50, 75)	[75, 100)
u_54_	[0, 25)	[25, 50)	[50, 75)	[75, 100)

**Table 15:** Correlation matrix of each evaluation indicator at the indicator layer.

	Correlations				
Evaluation Indicator	I	II	III	IV	LRS Rating
u_11_	−0.900	−0.829	−0.400	0.400	IV
u_12_	−0.493	−0.240	0.480	−0.255	III
u_13_	−0.733	−0.600	−0.200	0.200	IV
u_14_	−0.712	−0.500	−0.150	0.300	IV
u_21_	0.250	−0.250	−0.500	−0.667	I
u_22_	−0.167	0.250	−0.333	−0.444	II
u_23_	−0.324	0.080	−0.040	−0.360	II
u_24_	−0.467	−0.200	0.400	−0.273	III
u_31_	−0.867	−0.800	−0.600	0.400	IV
u_32_	−0.800	−0.700	−0.400	0.400	IV
u_33_	0.100	−0.100	−0.700	−0.775	I
u_34_	−0.400	−0.100	0.200	−0.308	III
u_41_	−0.573	−0.360	0.280	−0.179	III
u_42_	0.117	−0.130	−0.319	−0.440	I
u_43_	−0.867	−0.800	−0.600	0.400	IV
u_44_	−0.786	−0.780	−0.570	0.450	IV
u_45_	0.200	−0.200	−0.600	−0.733	I
u_51_	−0.419	−0.143	0.300	−0.280	III
u_52_	−0.600	−0.400	0.200	−0.143	III
u_53_	−0.533	−0.300	0.400	−0.222	III
u_54_	−0.467	−0.200	0.400	−0.273	III

**Table 16:** Correlation matrix of each factor category at the criteria layer.

	Correlations				
Evaluation Indicator	I	II	III	IV	LRS Rating
u_1_	−0.222	−0.181	−0.034	0.051	IV
u_2_	−0.031	0.001	−0.057	−0.125	II
u_3_	−0.113	−0.103	−0.083	0.029	IV
u_4_	−0.087	−0.078	−0.037	0.003	IV
u_5_	0.058	−0.028	0.037	−0.028	III

**Table 17:** Correlation matrix at the target layer.

	Correlations				
Evaluation Indicator	I	II	III	IV	LRS Rating
u	−0.113	−0.086	−0.040	−0.016	IV

## Data Availability

The data that has been used is confidential.

## Abbreviations

LRS	land reclamation suitability
BFSY	beam fabrication and storage yard
PYBFSY	PY beam fabrication and storage yard
AHP	analytic Hierarchy Process
MEA	matter-element analysis
KMO	Kaiser-Meyer-Olkin
ME	matter-element

## References

[1] Peng Y., Li T., Bao C., Zhang J., Xie G., Zhang H. (2023). Performance analysis and multi-objective optimization of bionic dendritic furcal energy-absorbing structures for trains. Int. J. Mech. Sci..

[2] Liang Y., Zhou K., Li X., Zhou Z., Sun W., Zeng J. (2020). Effectiveness of high-speed railway on regional economic growth for less developed areas. J. Transp. Geogr..

[3] Ren X., Chen Z., Wang F., Dan T., Wang W., Guo X., Liu C. (2020). Impact of high-speed rail on social equity in China: Evidence from a mode choice survey. Transp. Res. Part A Policy Pract..

[4] To W.M., Lee P.K.C., Yu B.T.W. (2020). Sustainability assessment of an urban rail system—The case of Hong Kong. J. Clean. Prod..

[5] Sun L., Li W. (2021). Has the opening of high-speed rail reduced urban carbon emissions? Empirical analysis based on panel data of cities in China. J. Clean. Prod..

[6] Ribeiro F.B., Nascimento F.A.C.d., Silva M.A.V.d. (2022). Environmental performance analysis of railway infrastructure using life cycle assessment: Selecting pavement projects based on global warming potential impacts. J. Clean. Prod..

[7] Pons J.J., Villalba Sanchis I., Insa Franco R., Yepes V. (2020). Life cycle assessment of a railway tracks substructures: Comparison of ballast and ballastless rail tracks. Environ. Impact Assess. Rev..

[8] Kaewunruen S., Sresakoolchai J., Peng J. (2020). Life Cycle Cost, Energy and Carbon Assessments of Beijing-Shanghai High-Speed Railway. Sustainability.

[9] Jang W., Lee S.K., Han S.H. (2018). Sustainable performance index for assessing the green technologies in urban infrastructure projects. J. Manag. Eng..

[10] Fu H., Niu J., Wu Z., Cheng B., Guo X., Zuo J. (2022). Exploration of public stereotypes of supply-and-demand characteristics of recycled water infrastructure—Evidence from an event-related potential experiment in Xi’an, China. J. Environ. Manag..

[11] Amirshenava S., Osanloo M. (2021). Mined land suitability assessment: A semi-quantitative approach based on a new classification of post-mining land uses. Int. J. Min. Reclam. Environ..

[12] Wang J., Zhao F., Yang J., Li X. (2017). Mining site reclamation planning based on land suitability analysis and ecosystem services evaluation: A case study in Liaoning province, China. Sustainability.

[13] Yu X., Mu C., Zhang D. (2020). Assessment of land reclamation benefits in mining areas using fuzzy comprehensive evaluation. Sustainability.

[14] Tang G., Zhang Z., Lv Q., Hao R., Wang K. (2020). Suitability Evaluation for Land Reclamation of Nonmetallic Mines in Xinjiang, China. Math. Probl. Eng..

[15] Krmac E., Djordjević B. (2017). An evaluation of train control information systems for sustainable railway using the analytic hierarchy process (AHP) model. Eur. Transp. Res. Rev..

[16] Bao F., Qiu J. (2021). Ecological Vulnerability Evaluation Model of Sichuan-Tibet Railway Based on Fuzzy Matter-element Analysis. IOP Conf. Ser.: Earth Environ. Sci..

[17] Wang Y., Anderson N., Torgashov E. (2021). Selection of geophysical methods based on matter-element analysis with analytic hierarchy process. Explor. Geophys..

[18] Sukarman S., Gani R.A. (2020). Ex-coal mine lands and their land suitability for agricultural commodities in South Kalimantan. J. Degrad. Min. Lands Manag..

[19] Yang R., Zhong C. (2022). Land Suitability Evaluation of Sorghum Planting in Luquan County of Jinsha River Dry and Hot Valley Based on the Perspective of Sustainable Development of Characteristic Poverty Alleviation Industry. Agriculture.

[20] Asmarhansyah A., Badayos R.B., Sanchez P.B., Cruz P.C.S., Florece L.M. (2017). Land suitability evaluation of abandoned tin-mining areas for agricultural development in Bangka Island, Indonesia. J. Degrad. Min. Lands Manag..

[21] Mandal V.P., Rehman S., Ahmed R., Masroor M., Kumar P., Sajjad H. (2020). Land suitability assessment for optimal cropping sequences in Katihar district of Bihar, India using GIS and AHP. Spat. Inf. Res..

[22] Liu Y., Guo Y., Li Y., Li Y. (2015). GIS-based effect assessment of soil erosion before and after gully land consolidation: A case study of Wangjiagou project region, Loess Plateau. Chin. Geogr. Sci..

[23] Ouyang W., Wu Y., Hao Z., Zhang Q., Bu Q., Gao X. (2018). Combined impacts of land use and soil property changes on soil erosion in a mollisol area under long-term agricultural development. Sci. Total Environ..

[24] Chen H., Li H., Wang Y., Cheng B. (2020). A comprehensive assessment approach for water-soil environmental risk during railway construction in ecological fragile region based on AHP and MEA. Sustainability.

[25] Singha S., Chatterjee S. (2022). Soil Pollution by Industrial Effluents, Solid Wastes and Reclamation Strategies by Microorganisms. Soil Health and Environmental Sustainability.

[26] Hao B., Kang L. (2014). Mine land reclamation and eco-reconstruction in Shanxi province I: Mine land reclamation model. Sci. World J..

[27] Guo L., Xu W.Y., Quek A., Wu D.Q. (2015). Leaching assessment of matrix land reclamation material. Environ. Geotech..

[28] Wang W., Liu H., Li Y., Su J. (2014). Development and management of land reclamation in China. Ocean Coast. Manag..

[29] Lendering K., Jonkman S.N., Van Gelder P., Peters D. (2015). Risk-based optimization of land reclamation. Reliab. Eng. Syst. Saf..

[30] Dhar A., Naeth M.A., Jennings P.D., Gamal El-Din M. (2020). Geothermal energy resources: Potential environmental impact and land reclamation. Environ. Rev..

[31] Gangwar P., Singh R., Trivedi M., Tiwari R.K. (2020). Sodic soil: Management and reclamation strategies. Environmental Concerns and Sustainable Development.

[32] Sokolov M., Semenov A., Spiridonov Y., Toropova E.Y., Glinushkin A. (2020). Healthy Soil—Condition for Sustainability and Development of the Argo-and Sociospheres (Problem-Analytical Review). Biol. Bull..

[33] Zhang L., Zhang S., Huang Y., Xing A., Zhuo Z., Sun Z., Li Z., Cao M., Huang Y. (2018). Prioritizing abandoned mine lands rehabilitation: Combining landscape connectivity and pattern indices with scenario analysis using land-use modeling. ISPRS Int. J. Geo-Inf..

[34] Worlanyo A.S., Li J. (2021). Evaluating the environmental and economic impact of mining for post-mined land restoration and land-use: A review. J. Environ. Manag..

[35] Favas P.J., Martino L.E., Prasad M.N. (2018). Abandoned mine land reclamation—Challenges and opportunities (holistic approach). Bio-Geotechnologies for Mine Site Rehabilitation.

[36] Yurchenko I.F., Bandurin M.A., Volosukhin V.A., Vanzha V.V., Mikheyev A.V. Reclamation measures to ensure the reliability of soil fertility. Proceedings of the International Scientific and Practical Conference “Agro-SMART-Smart Solutions for Agriculture” (Agro-SMART 2018).

[37] AlQahtany A.M., Dano U.L., Elhadi Abdalla E.M., Mohammed W.E., Abubakar I.R., Al-Gehlani W.A.G., Akbar N., Alshammari M.S. (2022). Land Reclamation in a Coastal Metropolis of Saudi Arabia: Environmental Sustainability Implications. Water.

[38] Bo M.W., Arulrajah A., Horpibulsuk S., Leong M. (2015). Quality management of prefabricated vertical drain materials in mega land reclamation projects: A case study. Soils Found..

[39] Shorokhova A., Novichikhin A., Yur’eva E. (2018). Socially Oriented Information Technology for Reducing the Environmental Impact of Mines and Metallurgical Enterprises. Steel Transl..

[40] Kong X., Wen L. The practice of reclaimed land be converted to arable land in China. Proceedings of the EGU General Assembly Conference Abstracts.

[41] Li F., Zhang S., Yang J., Bu K., Wang Q., Tang J., Chang L. (2016). The effects of population density changes on ecosystem services value: A case study in Western Jilin, China. Ecol. Indic..

[42] Valenzuela V.P.B., Esteban M., Onuki M. (2020). Perception of Disasters and Land Reclamation in an Informal Settlement on Reclaimed Land: Case of the BASECO Compound, Manila, the Philippines. Int. J. Disaster Risk Sci..

[43] Hendrychová M., Svobodova K., Kabrna M. (2020). Mine reclamation planning and management: Integrating natural habitats into post-mining land use. Resour. Policy.

[44] Janků J., Kučerová D., Houška J., Kozak J., Rubešová A. (2014). The evaluation of degraded land by application of the contingent method. Soil Water Res..

[45] Shan J., Li J. (2020). Valuing marine ecosystem service damage caused by land reclamation: Insights from a deliberative choice experiment in Jiaozhou Bay. Mar. Policy.

[46] Alary V., Aboul-Naga A., Osman M.A., Daoud I., Abdelraheem S., Salah E., Juanes X., Bonnet P. (2018). Desert land reclamation programs and family land dynamics in the Western Desert of the Nile Delta (Egypt), 1960–2010. World Dev..

[47] Tian L., Zhu J. (2013). Clarification of collective land rights and its impact on non-agricultural land use in the Pearl River Delta of China: A case of Shunde. Cities.

[48] Grydehøj A. (2015). Making ground, losing space: Land reclamation and urban public space in island cities. Urban Isl. Stud..

[49] Bisaro A. (2019). Coastal adaptation through urban land reclamation: Exploring the distributional effects. DIE ERDE–J. Geogr. Soc. Berl..

[50] Liu S., Zhang P., Lo K. (2014). Urbanization in remote areas: A case study of the Heilongjiang Reclamation Area, Northeast China. Habitat Int..

[51] Kovalenko P., Rokochinskiy A., Volk P., Turcheniuk V., Frolenkova N., Tykhenko R. (2021). Evaluation of ecological and economic efficiency of investment in water management and land reclamation projects. J. Water Land Dev..

[52] Saaty T.L. (1977). A scaling method for priorities in hierarchical structures. J. Math. Psychol..

[53] Cheng B., Chang R., Yin Q., Li J., Huang J., Chen H. (2023). A PSR-AHP-GE model for evaluating environmental impacts of spoil disposal areas in high-speed railway engineering. J. Clean. Prod..

[54] Sang K., Fontana G.L., Piovan S.E. (2022). Assessing railway landscape by ahp process with gis: A study of the yunnan-vietnam railway. Remote Sens..

[55] Marhavilas P.K., Tegas M.G., Koulinas G.K., Koulouriotis D.E. (2020). A joint stochastic/deterministic process with multi-objective decision making risk-assessment framework for sustainable constructions engineering projects—A case study. Sustainability.

[56] Feng L., Zhu X., Sun X. (2014). Assessing coastal reclamation suitability based on a fuzzy-AHP comprehensive evaluation framework: A case study of Lianyungang, China. Mar. Pollut. Bull..

[57] Shareef M.A., Ameen M.H., Ajaj Q.M. (2020). Change detection and GIS-based fuzzy AHP to evaluate the degradation and reclamation land of Tikrit City, Iraq. Geod. Cartogr..

[58] Alavi I. (2014). Fuzzy AHP method for plant species selection in mine reclamation plans: Case study sungun copper mine. Iran. J. Fuzzy Syst..

[59] Zhang K., Zheng W., Xu C., Chen S. (2019). An improved extension system for assessing risk of water inrush in tunnels in carbonate karst terrain. KSCE J. Civ. Eng..

[60] Cai W. (1999). Extension theory and its application. Chin. Sci. Bull..

[61] Doyle W.S. (2019). Surface mined land reclamation in Germany. Coal Surface Mining.

[62] Meng W., Hu B., He M., Liu B., Mo X., Li H., Wang Z., Zhang Y. (2017). Temporal-spatial variations and driving factors analysis of coastal reclamation in China. Estuar. Coast. Shelf Sci..

[63] Feng Y., Wang J., Bai Z., Reading L. (2019). Effects of surface coal mining and land reclamation on soil properties: A review. Earth-Sci. Rev..

[64] Wu J.-X., Guo X.-J., Xie Y.-Q., Zhang Z.-C., Tang H.-R., Ma Z.-J., Chen J.-B. (2021). Evolution of bubble-bearing areas in shallow fine-grained sediments during land reclamation with prefabricated vertical drain improvement. Eng. Geol..

[65] Lamprea-Pineda A.C., Connolly D.P., Hussein M.F. (2022). Beams on elastic foundations—A review of railway applications and solutions. Transp. Geotech..

[66] Qiu L., Zhang M., Zhou B., Cui Y., Yu Z., Liu T., Wu S. (2021). Economic and ecological trade-offs of coastal reclamation in the Hangzhou Bay, China. Ecol. Indic..

